# 0449. Surface EMG of extra-diaphragmatic muscles can assess muscle unloading during neurally adjusted ventilatory support

**DOI:** 10.1186/2197-425X-2-S1-O12

**Published:** 2014-09-26

**Authors:** P Somhorst, M van Mourik, AA Koopman, D Gommers

**Affiliations:** Erasmus MC, Adult Intensive Care, Rotterdam, Netherlands; University of Twente, Institute of Technical Medicine, Enschede, Netherlands

## Introduction

Neurally adjusted ventilatory assist (NAVA) is used to adapt mechanical ventilation to patient demand while unloading respiratory muscles. Titration methods are focused on sustained unloading of the diaphragm while maintaining a stable tidal volume [1]. It is hypothesized that the activity of accessory respiratory muscles can supply extra information about muscle unloading and patient comfort during NAVA ventilation.

## Objectives

To assess the extra-diaphragmatic muscle activity (EDMA) during 100%, 50% and 150% of titrated NAVA level.

## Methods

EDMA was measured in ventilated patients with mild ARDS. EDMA was defined as the amplitude of the combined surface EMG at the scalene and sternomastoid muscle (Dipha16, InBiolab, Groningen, The Netherlands). A baseline NAVA level (NAVA100) was titrated using the diaphragm activity (EAdi) response to changing NAVA levels, according to Brander et al. [1]. Patients were ventilated with NAVA100, NAVA50 (50% of NAVA100) and NAVA150 (150% NAVA100) for a period of 15 minutes.

## Results

Twenty-one patients were included. In six patients EDMA was absent during NAVA100, so NAVA titration was sufficient to unload accessory respiratory muscles. Fifteen patients (71%) showed EDMA during NAVA100. In seven patients (33%) EDMA increased at NAVA50 and decreased during NAVA150. In one patient, EDMA decreased at NAVA150. One patient only showed EDMA during NAVA50. In three patients EDMA decreased at NAVA50 or increased at NAVA150. Nine patients (43%) showed no change in EDMA after a change of NAVA level.

## Conclusions

Measurement of extra-diaphragmatic muscle activity using surface EMG during NAVA ventilation might be helpful in titration of ventilatory assist level in order to optimize patient´s comfort.
